# Gastric Adenocarcinoma of the Remnant Stomach Presenting With a Cystic Intra-abdominal Mass 11 Years After Roux-en-Y Gastric Bypass

**DOI:** 10.14309/crj.0000000000000810

**Published:** 2022-06-27

**Authors:** Gebran W. Khneizer, Neetu Mahendraker, Benjamin L. Bick

**Affiliations:** 1 Division of Gastroenterology and Hepatology, Saint Louis University School of Medicine, Saint Louis, MO; 2 Department of Internal Medicine and Geriatrics, Indiana University School of Medicine, Indianapolis, IN; 3 Indiana University Health Physicians, Indianapolis, IN; 4 Division of Gastroenterology and Hepatology, Indiana University School of Medicine, Indianapolis, IN

## CASE REPORT

Morbid obesity defined as a body mass index of ≥ 40 kg/m^2^ is associated with increased morbidity and all-cause mortality.^1^ Roux-en-Y gastric bypass (RYGB) is the desired surgical intervention because of its proven benefits.^1,2^ Morbid obesity is associated with increased risk of gastric cancer; however, the altered anatomy of the stomach after bypass is challenging for ongoing surveillance of the remnant stomach.^2^ To date, only 17 cases of gastric remnant malignancy after RYGB are reported.^2^ We present a rare case of gastric remnant adenocarcinoma 11 years after RYGB presenting with abdominal pain and weight loss due to a large intra-abdominal cystic mass abutting the gastric remnant.

**Figure 1.**
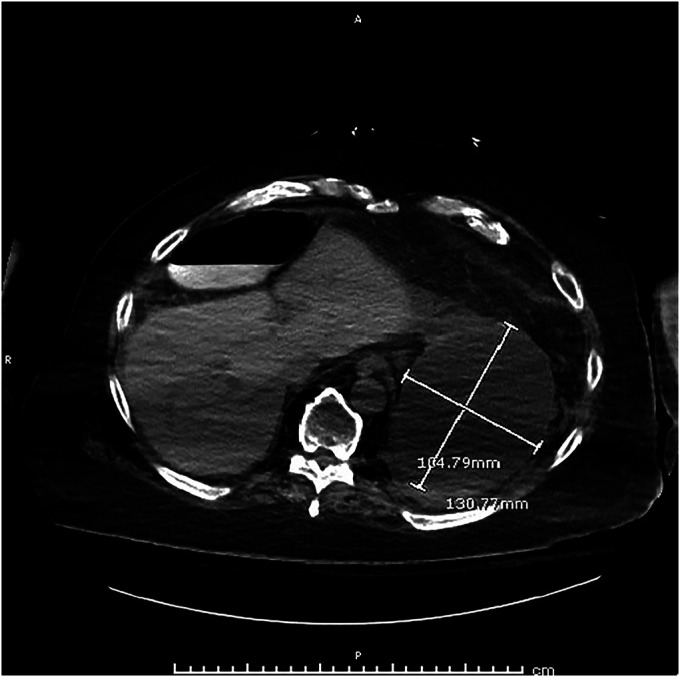
Axial images of the abdomen and pelvis show the cystic dilation.

**Figure 2.**
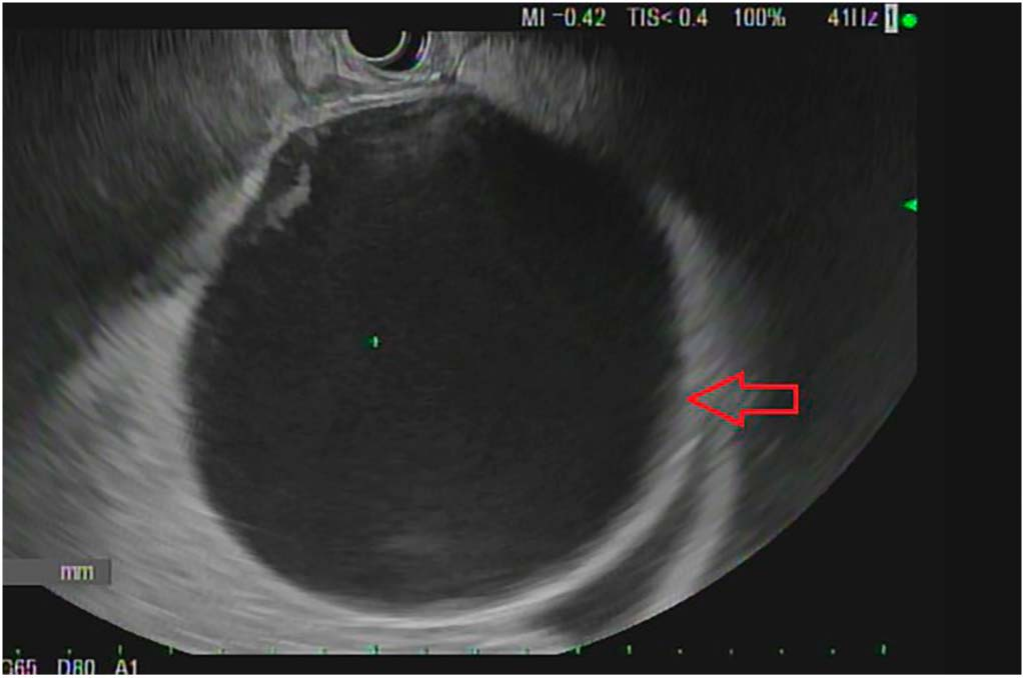
The cystic structure seen before fine-needle aspiration (FNA) (thick arrow).

**Figure 3.**
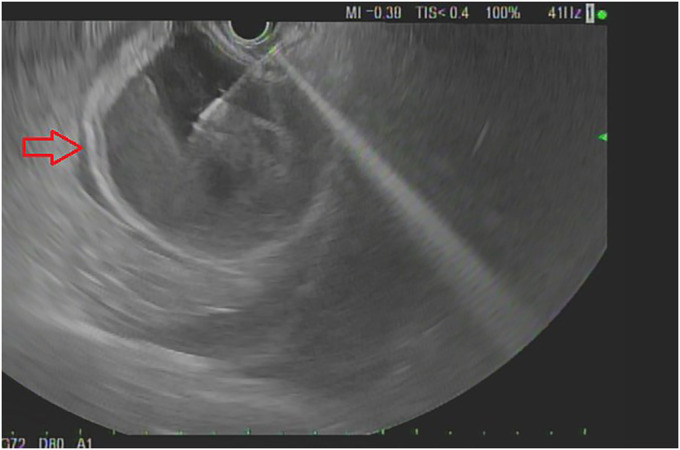
Endoscopic ultrasound (EUS) images revealing the wall layers of the cystic structure (thick arrow) is seen after cyst fluid fine-needle aspiration (FNA), which demonstrates that it is not a cyst but a gastrointestinal structure.

Abdominal computed tomography without contrast revealed a large cystic structure abutting the stomach and pancreas (Figure 1). An esophagogastroduodenoscopy was normal. An endoscopic ultrasound (EUS) revealed a single round anechoic lesion >100 mm in size in the peripancreatic and perigastric peritoneal space (Figure 2). Typical mucosal, submucosal, and muscular propria layers were noted within the wall of the cystic structure without communication to the excluded stomach (Figure 3). Fine-needle aspiration of fluid (Expect 19 Gauge Flex Needle [Boston Scientific, Marlborough, MA]) demonstrated 750 mL of brown fluid with unremarkable cytology and normal lipase and amylase levels. The carcinoembryonic antigen (CEA) level in the cystic fluid was markedly elevated at 2,358 ng/mL when compared with serum CEA of 76.6 ng/mL.

**Figure 4.**
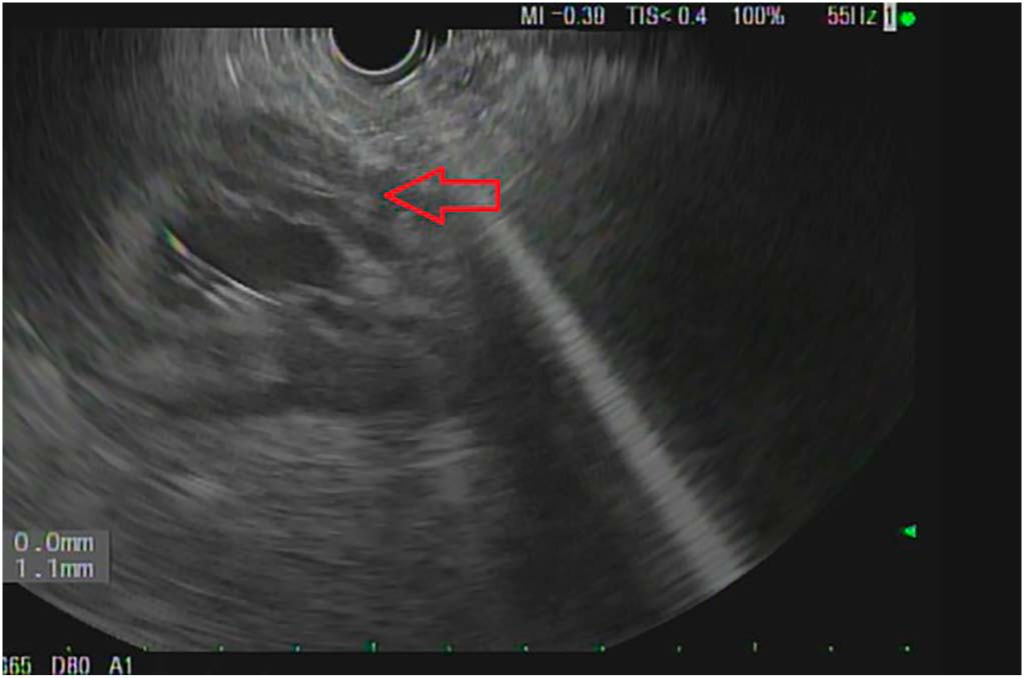
The area of the excluded stomach (thick arrow) with abnormal thickening of the stomach wall was biopsied revealing adenocarcinoma.

A second EUS performed because of recurrent abdominal distension showed a persistent cystic structure. Injection of contrast within the cystic structure demonstrated communication between the cyst and the excluded stomach. Fine-needle biopsy (Acquire 25 Gauge Needle [Boston Scientific]) of an area of the thickened gastric wall demonstrated poorly differentiated adenocarcinoma (Figure 4).

Diagnosis of gastric remnant malignancy is usually confirmed at a median of 9 years after RYGB (range is 1–22 years) with abdominal computed tomography scans.^2^ EUS and transgastric endoscopy may be considered as alternatives to double-balloon enteroscopy for tissue biopsy in the setting of technically difficult access of the gastric remnant.^3,4^ Prevention and surveillance methods including remnant gastrectomy proposed in regions with a high prevalence of gastric cancer remain controversial and not well established. More studies are needed to determine whether CEA can be considered for screening of the cancer in the excluded stomach in patients with a history of morbid obesity and RYGB.^5^

## DISCLOSURES

Author contributions: GW Khneizer and BL Bick conceived and drafted the work. N. Mahendraker designed and critically revised the work, and all authors gave final approval of the version to be published and agreed to be accountable for all aspects of the work. GW Khneizer is the article guarantor.

Financial disclosure: None to report.

Informed consent could not be obtained from the family of the deceased. All identifying information has been removed from this case report to protect patient privacy.
